# Therapeutic Properties of Bioactive Compounds from Different Honeybee Products

**DOI:** 10.3389/fphar.2017.00412

**Published:** 2017-06-28

**Authors:** Laura Cornara, Marco Biagi, Jianbo Xiao, Bruno Burlando

**Affiliations:** ^1^Dipartimento di Scienze della Terra, dell’Ambiente e della Vita, Università degli Studi di GenovaGenova, Italy; ^2^Unità Operativa di Biologia Farmaceutica, Dipartimento di Scienze Fisiche, della Terra e dell’Ambiente, Università degli Studi di SienaSiena, Italy; ^3^Institute of Chinese Medical Sciences, State Key Laboratory of Quality Research in Chinese Medicine, University of MacauTaipa, Macau; ^4^Dipartimento di Farmacia, Università degli Studi di GenovaGenova, Italy

**Keywords:** bee pollen, bee venom, beeswax, honey, propolis, royal jelly

## Abstract

Honeybees produce honey, royal jelly, propolis, bee venom, bee pollen, and beeswax, which potentially benefit to humans due to the bioactives in them. Clinical standardization of these products is hindered by chemical variability depending on honeybee and botanical sources, but different molecules have been isolated and pharmacologically characterized. Major honey bioactives include phenolics, methylglyoxal, royal jelly proteins (MRJPs), and oligosaccharides. In royal jelly there are antimicrobial jelleins and royalisin peptides, MRJPs, and hydroxy-decenoic acid derivatives, notably 10-hydroxy-2-decenoic acid (10-HDA), with antimicrobial, anti-inflammatory, immunomodulatory, neuromodulatory, metabolic syndrome preventing, and anti-aging activities. Propolis contains caffeic acid phenethyl ester and artepillin C, specific of Brazilian propolis, with antiviral, immunomodulatory, anti-inflammatory and anticancer effects. Bee venom consists of toxic peptides like pain-inducing melittin, SK channel blocking apamin, and allergenic phospholipase A2. Bee pollen is vitaminic, contains antioxidant and anti-inflammatory plant phenolics, as well as antiatherosclerotic, antidiabetic, and hypoglycemic flavonoids, unsaturated fatty acids, and sterols. Beeswax is widely used in cosmetics and makeup. Given the importance of drug discovery from natural sources, this review is aimed at providing an exhaustive screening of the bioactive compounds detected in honeybee products and of their curative or adverse biological effects.

## Introduction

Honeybees are social hymenopteran insects belonging to the genus *Apis*, characterized by the production and storage of honey and other substances potentially useful to humans. Two domesticated species are currently known, i.e., the western *A. mellifera*, native to Europe, Asia and Africa, and introduced into America, and the eastern *A. cerana*, distributed in southern and southeastern Asia.

Honey is the main and most widely appreciated honeybee product. It derives from digestive processing of nectar foraged from flowers, and is stored in honeycomb cells. Honey is generally marketed for its nutritive properties, but has been also used since the antiquity as a folk remedy, while in recent times it has been introduced as a pharmaceutical aid and in clinical practice (Molan, 1999). Other products derive from honeybee gland secretions and different botanical materials, either alone or variously mixed together. These substances include royal jelly, beeswax, propolis, bee pollen, and bee venom. All of them have been used by humans since ancient times for nutritional and curative purposes.

Several biological properties have been detected in honeybee products by a wide series of scientific studies, while different reviews have been dedicated to summarize therapeutic properties and uses as nutraceutical, pharmaceutical and cosmetic ingredients (Viuda-Martos et al., 2008; Burlando and Cornara, 2013). Various attempts at introducing some of these products in clinical settings have been made, but their pharmacological and medicinal standardization is made difficult by the high chemical variability, depending on honeybee varieties and botanical sources. However, different molecules or classes of compounds have been isolated and some of them have also been pharmacologically characterized, suggesting the importance of honeybee products for drug discovery from natural sources. Given the importance of this latter field in the search for new remedies against problematic diseases, this review is aimed to provide an exhaustive screening of the bioactive compounds detected in honeybee products and of their curative or adverse biological effects. Literature data were collected in Scopus, Web of Science, PubMed, Google Scholar, https://clinicaltrials.gov, and Espacenet databases. General medicinal application of honeybee products has been covered, while more in depth search on bioactivities and putative therapeutic effects has been conducted on the major constituents of these products.

## Honey

Honey is produced by foraging bees that collect flower nectar and process it through repeated digestion and regurgitation. Stomach acidic pH, together with invertase, diastase and amylase enzymatic activities, give rise to a supersaturated aqueous solution composed by 80% sugars, mainly fructose and glucose, with minor amounts of sucrose, maltose, and other complex sugars.

Most nitrogen is present in amino acids and peptides. Proline is the most abundant amino acid, followed by glutamic acid, alanine, phenylalanine, tyrosine, leucine, isoleucine, and other minor ones. Honey also contains low amounts of protein, usually 0.1÷1.5% in the western honeybee *A. mellifera*, and 0.1÷3.0% in the Asiatic honeybee *A. cerana*. Most abundant peptides are defensin-1 and royal jelly protein (MRJP) isoforms, while major enzymes include glucose oxidase, diastase (amylase), α-glucosidase, catalase, and acid phosphatase (Kubota et al., 2004; Di Girolamo et al., 2012; Chua et al., 2015). Data about honey proteome are limited, but it is arguable that each honey type has its own peptide pattern, consisting of ubiquitous components and a variable set of minor elements, possibly including plant peptides.

Honey has an average pH of 3.9, mainly due to the presence of about 0.57% organic acids, predominantly gluconic acid originating from glucose oxidase activity, and citric acid. Small amounts of vitamins are also present, especially vitamin B complex due to pollen grains, and ascorbic acid. Minerals range between 0.04 and 0.2%, reflecting the mineral content of soils where source plants grow. Potassium is the major element, accounting for about one third of total mineral content.

Different plant compounds are present in honey at low concentrations and in variable amounts depending on the botanical species visited by bees and the climate of the area from which nectar is harvested. Aroma compounds are probably the most diversified fraction, as testified by the finding of over 500 volatile compounds in different types of honey. Phenolics are the most abundant phytochemicals, usually ranging from 50 to 500 mg/kg (Ramanauskiene et al., 2012; da Silva et al., 2016).

### Antioxidant Activity

It is generally accepted that phenolics are important contributors to the antioxidant capacity of honey. Given that phenolic composition is greatly variable with respect to floral origin, honey is expected to show a wide range of antioxidant power (Gheldof et al., 2002; Petretto et al., 2015).

### Antimicrobial Activity

Honey is known to contrast the growth of various microorganisms. This kind of effect has been a main attractive feature for honey application in clinical medicine, as testified by the development and marketing of γ-irradiated, Manuka medical grade honey. For this honey, a scale of antibacterial activity has been defined, known as Unique Manuka Factor (UMF), representing equivalents of a phenol solution yielding a certain inhibition in a radial diffusion assay on *Staphylococcus aureus* (Allen et al., 1991).

The role of specific honey antibacterial factors has been studied by sequential neutralization, showing that the mechanisms of action are complex and variable in different honey types. Basic antibacterial factors are low water activity and low pH. In addition, honey generally contains glucose oxidase added by bees, which by low dilution converts glucose into H_2_O_2_ and gluconic acid. However, measurements of minimum inhibitory concentration and DNA degradation on *Escherichia coli* and *Bacillus subtilis* has shown that honey H_2_O_2_ concentrations would not fully account for bacterial growth inhibition, suggesting that other honey components should be involved (Brudzynski et al., 2011). Two major non-peroxide antibacterial factors are methylglyoxal (MGO) in manuka honey and defensin-1 in Revamil source (RS) honey, which also contains MGO, but to a much lower extent than manuka honey. RS honey is the source product for Revamil^®^ medical grade honey (Kwakman and Zaat, 2012).

Manuka honey derives from the nectar of the manuka myrtle *Leptospermum scoparium*, growing in southeast Australia and New Zealand. In this honey, high amounts of MGO are formed from dihydroxyacetone, present at high levels in manuka nectar, through a non-enzymatic process occurring during honey storage. The very high content of MGO, up to about 1,500 mg/kg, makes this honey able to kill various bacterial strains, including methicillin-resistant *S. aureus* (Mavric et al., 2008). However, other unidentified compounds besides MGO are likely to contribute to antibacterial activity, since it has been experimentally shown that MGO neutralization affects honey activity against *S. aureus* and *B. subtilis*, but not against *E. coli* and *Pseudomonas aeruginosa* (Kwakman et al., 2011).

Defensin-1 is an immunoactive peptide secreted by the honeybee hypopharyngeal gland and ultimately transferred to honey. The peptide has been detected in RS honey and is responsible for antibacterial activity against Gram-positive bacteria like *Bacillus* spp. Conversely, defensin-1 is not effective against *S. aureus* if used alone, but is essential for honey activity against this bacterial species, suggesting a possible synergistic interaction with other honey components. Peptide-dependent antibacterial activities have been found in other honey types besides RS honey, possibly depending in some cases on the presence of defensin-1. Other evidence for synergistic interactions have been detected in the effect of RS honey on *E. coli* and *P. aeruginosa*, requiring both H_2_O_2_ and MGO, and on vancomycin-resistant *Enterococcus faecium*, requiring H_2_O_2_ in combination with either MGO or defensin-1 (Kwakman and Zaat, 2012).

A specific study has been devoted to compare the antibacterial mechanisms of action of Manuka and RS honey, showing defensin-1 and H_2_O_2_ as major antibacterial factors of RS honey. Conversely, in manuka honey these factors have not been observed, while the antibacterial activity has been found to depend on MGO and other unidentified factors (Kwakman et al., 2011). However, another study has shown that the apparent lack of defensin-1 in manuka would be due to an inactivation of the peptide caused by MGO-induced modifications (Majtan et al., 2012).

Other antibacterial factors isolated from honey are glycoproteins with high-mannose N-glycans. These proteins have displayed agglutinating and bactericidal activity on different clinical isolates of multi drug resistant strains, such as methicillin-resistant *S. aureus* MRSA, *P. aeruginosa*, *Klebsiella pneumoniae*, vancomycin-resistant enterococci (VRE), and extended-spectrum β-lactamase-producing (ESBL) *Proteus mirabilis*, and *E. coli*. Q-TOF-MS analysis has shown extensive homology of these peptides with the MRJP-1 precursor, which harbors three antimicrobial jelleins typical of royal jelly. These data indicate a role for high-mannose structures in the antibacterial activity of honey glycoproteins (Brudzynski and Sjaarda, 2015; Brudzynski et al., 2015).

Fractionation of an n-hexane extract of *Citrus* goldcrest honey has led to the identification of a complex fraction with inhibitory activity against a clarithromycin/metronidazole-resistant *Helicobacter pylori* strain. This fraction contains mostly acetic acid, 2,3-dihydro-3,5-dihydroxy-6-methyl-4H-pyran-4-one, 2-propanone, butanal, 1,3-benzenediamine, propanenitrile, 2-furanmethanol, propanoic acid, 1,3-butanediol, 1-(1-cyclopentenyl)-1-propanol, and 5-hydroxymethylfurfural (Manyi-Loh et al., 2012). However, the specific role of each single compound, or the presence of combinatory effects, has not been ascertained.

Honey is also known for antimycotic effects, e.g., against non-pathogenic *Aureobasidium pullulans* and *Cladosporium cladosporioides*, and pathogenic *Candida parapsilosis*, *C. tropicalis*, and *Rhodotorula* sp. (Kuncic et al., 2012; Moussa et al., 2012). Evidence has been collected that flavonoids like quercetin, kaempferol, chrysin, galangin, and apigenin may be involved in honey activity against *C. albicans* (Candiracci et al., 2011).

### Antiparasitic Activity

Peptides from *Ziziphus* sp. honey with molecular masses ranging from 2 to 200 kDa, separated by size-exclusion chromatography, have shown antiprotozoal activity against the intestinal parasite *Giardia lamblia* (Mohammed et al., 2015). In addition, three different honeys from *Plectranthus*, *Ziziphus*, and acacia, have been found to possess nematicidal activity against *Caenorhabditis elegans*, which has been correlated to the presence of an unidentified glycoconjugate of about 5 kDa (Sajid and Azim, 2012).

### Anti-inflammatory Activity

An extract from Italian multifloral honey, containing the flavonoids daidzein, apigenin, genistin, luteolin, kaempferol, quercetin, and chrysin, as major components, has inhibited the release of pro-inflammatory TNF-α and IL-1β from LPS-stimulated N13 microglia cells (Candiracci et al., 2012). Given a role of neuroinflammation in neurodegenerative diseases, these data confirm possible use of the honey flavonoid fraction in contrasting disorders like Alzheimer or Parkinson.

Immunomodulatory effects have been reported also for honey proteins. MRJP-3 has been found to suppress IL-2, IL-4, and IFN-γ production by antigen-stimulated T cells (Okamoto et al., 2003). Glycopeptides and glycoproteins isolated from *Ziziphus* honey, ranging from 2 to 450 kDa, have inhibited ROS release by zymosan-activated human neutrophils and murine macrophages, NO production and phagocytosis by LPS-activated murine macrophages, and production of TNF-α by human monocytic cells (Mesaik et al., 2015).

The honey protein apalbumin-1, aka MRJP-1, has been found to block the mannose receptors of human phagocytic cells, thereby inhibiting phagocytic activities. Such inhibitory effect seems enhanced in MGO-containing honey, due to apalbumin glycation (Molan and Rhodes, 2015).

### Antidiabetic Activity

Honey has been found to reduce blood glucose in animal models and in patients with impaired glucose tolerance or diabetes, though clinical studies have not provided conclusive evidence. Fructose is a potential antidiabetic agent, while the presence in honey of a balanced mix of fructose and glucose could play an additional role in this kind of effect, since the two sugars are known to act synergistically in promoting liver glucose metabolism (Erejuwa et al., 2012).

It has been shown that non-digestible, dietary oligosaccharides, such as fructooligosaccharides, galactooligosaccharides, and lactulose, have a preventive role against obesity, insulin resistance, and diabetes mellitus, by acting as prebiotics on the intestinal flora. Given that honey contains oligosaccharides, it has been hypothesized that these compounds might contribute to honey prebiotic effects, and therefore, that they could be linked to honey antidiabetic, antihyperlipidemic and hepatoprotective virtues (Erejuwa et al., 2014). However, experimental evidence in support of such assumption is lacking.

### Wound Healing

Honey has long been known for its healing capacity on wounds and burns (Molan, 2006; Vandamme et al., 2013). Revamil^®^ medical grade honey has been specifically developed for this purpose, while another medical-grade, wound-dressing product is Surgihoney^TM^, consisting of engineered honey with enhanced antimicrobial power (Al-Waili et al., 2011).

Antimicrobial activity is considered the most important factor for honey wound healing. However, various studies indicate that honey may also specifically act on skin cells involved in the wound healing process (Majtan et al., 2010; Ranzato et al., 2012). Various data have been collected on honey immunomodulatory properties, which can at least partially explain the ability of promoting wound healing. Even though some authors have argued that honey immunostimulatory effects may derive from bacterial lipopolysaccharide contamination, possible immunomodulatory honey constituents have been isolated. A 5.8 kDa constituent from manuka honey stimulates TNF-α production by macrophages via toll-like receptors (Tonks et al., 2007), while MRJP-1 induces TNF-α and metalloproteinase 9 (MMP-9) expression in keratinocytes (Majtan et al., 2010). Kanuka honey from *Kunzea ericoides*, a close relative of the manuka myrtle *L. scoparium*, contains type II arabinogalactans of plant origin that have been shown to promote TNF-α production by monocytic cell lines differentiated into macrophage by phorbol myristate acetate (Gannabathula et al., 2012). An aqueous extract rich in phenolics, obtained from fir honeydew honey, has been found to inhibit TNF-α-induced MMP-9 production by human keratinocytes, with a possible role for kaempferol and apigenin (Majtan et al., 2013). Such a result is apparently in contrast with another study reporting that different types of honey stimulate MMP-9 expression in the same cells (Ranzato et al., 2012), but in this latter case, whole honey has been used, possibly resulting in a dominant stimulatory effect due to other components, like for instance MRJPs.

### Anticancer Activity

Inhibitory effects of honey on various kinds of cancer have been studied both *in vitro* and in animal models (Erejuwa et al., 2014). Polyphenols are known to possess chemopreventive properties, and accordingly, it has been shown that honey with higher phenolic charge is more potent in inhibiting cancer cell proliferation (Jaganathan and Mandal, 2009). Various polyphenols occurring in honey have been studied singularly for their mechanisms of action on cancer models, including caffeic acid and its phenyl esters, caffeoylquinic acid derivatives, rosmarinic acid and derivatives, ellagic acid, as well as the flavonoids chrysin, luteolin, acacetin, fisetin, myricetin, wogonin, apigenin, hesperidin, galangin, quercetin, kaempferol, pinobanksin, and pinocembrin (Jaganathan and Mandal, 2009; Abubakar et al., 2012). However, direct evidence that these compounds or their combinations are responsible for honey anticancer activity is lacking.

A unique trihydroxyketone (E-4-(1,2,4-trihydroxy-2,6,6-trimethylcyclohexyl)-but-3-en-2-one) from thyme honey, endowed with antibacterial activity, has been shown to induce apoptosis on PC-3 prostate cancer cells. Such an effect has been put in relationship with the inhibition of p65 NF-KB phosphorylation and IL-6 secretion, but these inhibitory activities have been found at concentrations ranging between 1 and 100 μM, whereas apoptosis has been observed with the 100 μM dose only (Kassi et al., 2014).

### Adverse Effects

Various occurrences of toxic compounds in honey have been reported, such as polycyclic diterpene grayanotoxins in honey from rhododendron plants like *R. luteum* and *R. ponticum*. This kind of honey is known as “mad honey” since it may produce severe neural intoxication up to fatal emergency, especially in the eastern Black Sea region of Turkey. Grayanotoxins are known to affect voltage-dependent Na^+^ channel gating. Possibly due to this kind of action, and despite its toxicity, mad honey is used as folk medicine for hypertension, sexual dysfunction, and other ailments (Koca and Koca, 2007; Silici and Atayoglu, 2015).

Plants like Boraginaceae, Asteraceae, and Fabaceae produce pyrrolizidine alkaloids that are not toxic *per se* but are converted into harmful pyrrolic metabolites by liver after honey ingestion. The presence of these alkaloids in typical honey botanical sources, make these compounds a potential hazard for honey consumers (Edgar et al., 2002).

Cases of intoxication from honey consumption in New Zealand, characterized by delirium, seizures, and memory loss, have been related to honey contamination by the neurotoxic sesquiterpene lactones tutin and hyenanchin. These oxygenated sesquiterpene picrotoxanes, targeting GABAergic and glycinergic receptors, are ingested by honeybees collecting honeydew produced by passionvine hoppers (*Scolypopa australis*) feeding on sap of the poisonous shrub tutu (*Coriaria* spp.) (Fields et al., 2014; Larsen et al., 2015). Other plant secondary metabolites, which are found in honey and could induce deleterious effects to humans, include hyoscyamine and hyoscine from Solanaceae, saponins from Sapindaceae, strychnine and gelsemine from Gelsemiaceae, oleandrin and oleandrigenin from Apocynaceae (Islam et al., 2014).

Besides phytochemicals, honey can also be contaminated by environmental pollutants, like heavy metals, pesticides, and antibiotics. Moreover, prolonged honey storage or heating may give rise to Maillard reaction products, such as the furans 5-hydroxymethylfurfural from hexoses and furfural from pentoses (Islam et al., 2014).

## Royal Jelly

Royal jelly is a secretion of honeybee hypopharynx and mandibular salivary glands. It is a white-yellowish, gelatinous, acidic colloid, containing about 67% water (w/w), 16% sugar, 12.5% protein and amino acids, and 5% fat, with considerable variability among different sources. Minor royal jelly constituents include enzymes, vitamins, phenolics, and minerals (Melliou and Chinou, 2005).

Proteins are the most abundant dry matter fraction, consisting for more than 80% of soluble glycoproteins named major royal jelly proteins (MRJPs), of which nine members have been described. These proteins are encoded by a family of genes arranged in tandem array, sharing a common ancestor with the Yellow protein family genes (Drapeau et al., 2006). MRJP-1 is the most abundant one and consists of monomeric and oligomeric forms. The oligomer, ranging between 350 and 420 kDa, can be separated into 55 and 5 kDa units, identified as MRJP-1 monomers and the 5 kDa protein apisimin, which is believed to act as subunit linker. MRJP-2, MRJP-3, MRJP-4 and MRJP-5 are glycoproteins ranging between 49 and 80 kDa (Tamura et al., 2009).

The lipid fraction mostly consists of medium chain fatty acids, terminally and/or internally hydroxylated, with terminal mono- or dicarboxylic acid functions, either saturated or monounsaturated at the 2-position. Major constituents are the 10-carbon atoms fatty acids *trans*-10-hydroxy-2-decenoic acid (10-HDA), unique to royal jelly, and 10-hydroxydecanoic acid. Sterols are also present in minor amounts (Li et al., 2013).

Royal jelly is fed until the 3rd day of life to larvae developing into female workers and male drones, or until the end of the larval period to selected individuals developing into queens. Moreover, it is an exclusive food for adult queens throughout their life (Fujita et al., 2013). The induction of larval development into queen has been ascribed to major royal jelly proteins (MRJPs) and a 57-kDa protein known as royalactin (Kamakura, 2011; Buttstedt et al., 2013), although contrary opinions have been raised against the alleged role of this latter (Buttstedt et al., 2016).

Royal jelly has been used since ancient times in traditional medicine, especially in Asiatic apitherapy, but also in the ancient Egypt. It is currently used in the pharmaceutical and cosmetic fields, and marketed as an over-the-counter functional food. Various studies have reported antimicrobial activities of royal jelly against bacteria, fungi, and viruses, while in animal models, hypotensive, antitumour, antihypercholesterolemic, and anti-inflammatory activities have been observed (Ramadan and Al-Ghamdi, 2012). Moreover, antidiabetic properties, positive effects on benign prostatic hyperplasia, and wound healing of diabetic foot ulcers have been verified in clinical trials (Siavash et al., 2015; Khoshpey et al., 2016). Most studies concern crude royal jelly or protein and lipid subfractions, but in several cases the activity of singular compounds has been ascertained.

### Antioxidant Activity

Small peptides consisting of 2–4 amino acid residues have been reported to possess strong antioxidant activity. Most active ones have tyrosine residues at the C-terminal, allowing hydroxyl radical and H_2_O_2_ scavenging activities (Guo et al., 2009).

### Antimicrobial Activity

The above-mentioned jelleins are four 8–9 amino acid peptides, of which jellein-I, -II, and -IV are cleavage products of MRJP-1. Antimicrobial assays conducted against the gram-positive *S. aureus*, *S. saprophyticus*, and *B. subtilis*, the gram negative *E. coli*, *E. cloacae*, *K. pneumoniae*, and *P. aeruginosa*, and the yeast *C. albicans* have shown that jellein-I and -II have broad-spectrum activity, jellein-III is less active, and jellein-IV has no antimicrobial effect (Fontana et al., 2004).

Royalisin is a 51 amino acid peptide, homologous to the haemolymph defensin-1, with antibacterial activity against various gram-positive strains, including *Staphylococcus*, *Streptococcus*, *B. subtilis*, *Micrococcus luteus*, *Sarcina lutea, Clostridium*, *Corynebacterium*, *Lactobacillus helveticus*, *Paenibacillus larvae*, and *Leuconostoc*, while no inhibition has been observed against the gram negative *E. coli* and *Serratia marcescens*. Antifungal activity against *Botrytis cinerea* has also been reported for royalisin (Fujiwara et al., 1990; Bachanova et al., 2002). In addition, a variant of MRJP-2, known as apalbumin 2a, has been found to inhibit the growth of *P. larvae*, *B. subtilis*, and *E. coli* (Bilikova et al., 2009).

Royal jelly carboxylic acids are known to collectively exert antimicrobial properties against gram-positive, gram-negative bacteria, and fungi. 10-HDA has been identified as a particularly strong antibacterial, especially against *B. subtilis*, *S. aureus*, and *E. coli* (Alreshoodi and Sultanbawa, 2015). Moreover, the compound has been shown to interfere with the adherence to cell surfaces of the oral pathogen *S. mutans*, by interfering with the expression of the glucosyltransferases gtfB and gtfC (Yousefi et al., 2012). Strong antifungal activity against *C. albicans*, *C. tropicalis*, and *C. glabrata* has been reported for sebacic acid (Melliou and Chinou, 2005).

### Anti-inflammatory Activity

In a study on royal jelly potential for digestive trait diseases, 10-HDA has been found to protect rats from experimentally induced gastric ulcer (Fang et al., 1994). A mechanism putatively linked to 10-HDA anti-inflammatory effect is the inhibition of LPS-induced NF-κB activation observed in the murine macrophage cell line RAW264 (Sugiyama et al., 2012).

10-HDA and 4-hydroperoxy-2-decenoic acid ethyl ester have been shown to inhibit histone deacetylase activity, thereby enhancing the expression of extracellular SOD release by leukemia THP-1 cells, and suggesting therapeutic potential against atherosclerosis (Makino et al., 2016). Histone deacetylase inhibition by 10-HDA is thought to reactivate the expression of epigenetically silenced genes in mammalian cells, leading to the hypothesis that a similar effect could be at the basis of caste switching in bees (Spannhoff et al., 2011). Modifications of histone acetylation have also emerged from a study showing 10-HDA inhibition of fibroblast-like synoviocytes from rheumatoid arthritis patients, suggesting potential therapeutic effects against chronic inflammation degenerative disease (Wang et al., 2015).

### Immunomodulatory Activity

Experimental evidence has been collected arguing for royal jelly immunomodulatory properties. A 57 kDa and 350-kDa royal jelly proteins, purportedly monomeric and oligomeric MRJP-1, have been reported to stimulate the proliferation of *in vitro* cultured hepatocytes and monocytes, respectively (Kamakura et al., 2001; Kimura et al., 2003). As a confirmation, an *in vitro* study has shown that the MRJP-1 oligomer, but not MRJP-2 or MRJP-3, induces the proliferation of the human lymphoid cell line Jurkat (Moriyama et al., 2015). MRJP-1 and MRJP-2 have been found to exert immunostimulatory and proinflammatory activities by stimulating cytokine release, such as TNF-α, from macrophages (Simuth et al., 2004; Majtan et al., 2006). In contrast, MRJP-3 has been reported to suppress interleukin production by T cells both *in vitro* and *in vivo*, reconducible to antiallergic properties (Okamoto et al., 2003).

Various immunomodulatory activities have been reported for 10-HDA, including reduced T cell proliferation, inhibition of interleukin-12 production by spleen dendritic cells, and block of LPS- and IFN-β-induced NO production in macrophages (Gasic et al., 2007; Sugiyama et al., 2013). In another study, a biphasic behavior of 10-HDA on human monocyte-derived dendritic cells has been found, resulting in Th1 response stimulation and Th2 downregulation at 50 μM, and repression of both Th1 and Th2 at 500 μM (Mihajlovic et al., 2013).

Another hydroxyl fatty acid, 3,10-dihydroxydecanoic acid, has been reported to stimulate the maturation of human monocyte-derived dendritic cells and their Th1 polarizing capability, suggesting a reinforcement of antitumour and antiviral immunity (Dzopalic et al., 2011). The immunomodulatory properties of royal jelly lipids suggest their possible use in interventions on autoimmune diseases.

### Metabolic Syndrome Preventing Activity

The royal jelly proteins MRJP-1, MRJP-2, and MRJP-3 have been shown to possess bile acid-binding properties. The most active one is MRJP-1, which in rats has increased fecal bile acids and cholesterol excretion, and has powered hepatic cholesterol catabolism (Kashima et al., 2014). In a study aimed at disclosing royal jelly antihypertensive mechanisms, MRJP-1 has been transfected into vascular smooth muscle cells, leading to a reduction of contraction, migration and proliferation (Fan et al., 2016).

A study conducted both *in vitro* on L6 myotubes, and *in vivo* on mice, has demonstrated that 10-HDA enhances insulin-independent muscle glucose uptake via AMP-activated protein kinase activation and GLUT4 translocation to the plasma membrane (Takikawa et al., 2013). Moreover, this fatty acid has been found to improve hyperlipidemic condition in a rat model (Xu et al., 2002).

### Anti-aging Activity

Royal jelly is known to extend the lifespan of honeybees. This property is at least in part due to royalactin, which has been found to induce such effect in other insect species, like *Drosophila melanogaster*, as well as in non-insect species, like the nematode *C. elegans* (Detienne et al., 2014). Moreover, also10-HDA has been found to increase longevity and confer thermal and oxidative stress tolerance to *C. elegans*, possibly through dietary restriction and TOR kinase signaling (Honda et al., 2015).

Royal jelly has estrogen-like effects, which in different studies have been ascribed to the ability of different lipids to act as weak activators of estrogen receptors. These constituents include 10-HDA, *trans*-2-decenoic, 10-hydroxydecanoic, 3,10-dihydroxydecanoic, and sebacic acids, and in addition the steroid 24-methylenecholesterol (Suzuki et al., 2008; Moutsatsou et al., 2010). These results have been proposed as a pharmacological basis for an anti-menopause use of royal jelly.

10-HDA has been shown to increase collagen synthesis and production of the collagen promoting factor, transforming growth factor β1, in human skin fibroblasts. Such an effect is thought to mediate royal jelly skin protection against UVB-induced photoaging (Koya-Miyata et al., 2004; Park et al., 2011). In addition to promoting collagen synthesis, 10-HDA has been found to inhibit the release of the MMP-1 and MMP-3 from rheumatoid arthritis synovial fibroblasts, possibly through downregulation of the pathway involving JNK/p38 MAP kinases and AP-1 transcription factor (Yang et al., 2010), and of the MMP regulator connective tissue growth factor (Wang et al., 2012). 10-HDA has also prevented UVA-induced JNK/p38 activation and MMP-1 and MMP-3 upregulation in fibroblasts (Zheng et al., 2013).

Such a complex of effects suggest skin dermal protection and antirheumatoid activity, but in most cases concentrations ranging around the millimolar level have been used, apparently with low clinical feasibility. However, a registered, synthetic 10-HDA counterpart, known as Hydroxydecine^®^, has been shown to activate keratinocyte differentiation *in vitro*, to restore skin barrier function in skin equivalents, and to improve UV-induced xerosis in human volunteers (Duplan et al., 2011).

### Neuromodulatory Activity

10-HDA and 10-hydroxydecanoic acid have been shown to act as potent agonists of the human TRPA1 and TRPV1 receptors (Terada et al., 2011). 10-HDA has stimulated neuron differentiation from rat embryo neural stem cells, possibly acting like the ω-3 docosahexaenoic acid, an essential diet component that is known to promote neurogenesis in the central nervous system. Docosahexaenoic acid is reputed essential for brain development and function and has shown positive effects in a rat Parkinson’s model, suggesting similar potentials for 10-HDA, which in addition could cross more easily the blood-brain barrier due to its smaller molecule (Hattori et al., 2007). Neurogenerative potentials of royal jelly fatty acids are also suggested by a study on synthetic, medium-chain fatty acids, in which 2-decenoic acid ethyl ester, a derivative of the royal jelly 2-decenoic acid, has promoted functional recovery in a rat model of spinal cord injury (Hirakawa et al., 2010).

### Adverse Effects

Similarly to honey, environmental contaminants can also be present in royal jelly. Most common ones are pesticides belonging to organochlorines, organophosphorus and carbamates, which are generally below Minimal Risk Level. However, in some cases the highly toxic, zero-tolerance chloramphenicol has been found (Bogdanov, 2006).

Royal jelly consumption can occasionally lead to contact dermatitis, asthma and anaphylaxis, while MRJP-1 and MRJP-2 have been identified as major allergens (Rosmilah et al., 2008).

## Propolis

Propolis is a resinous substance that foraging bees produce by collecting resin from buds and other plant tissues and then mixing it with wax and pollen to have a malleable, compact substance that they use as hive repairing material and sanitizer (Sun et al., 2015). The chemical composition of propolis is dramatically dependent on its geographical and floral origins. Raw propolis generally contains more than 300 different compounds, mostly consisting of triterpenes (50% w/w), waxes (25–30%), volatile mono- and sesquiterpenes (8–12%), giving propolis its typical resinous odor, and phenolics (5–10%) (Huang et al., 2014). European and Asian propolis contain simple phenolic acids (Bankova et al., 2002), while lignans are main compounds in tropical propolis (Petrova et al., 2010). Caffeic acid phenethyl ester (CAPE) is a major active found in European, Asian and American propolis (Omene et al., 2013). Brazilian green propolis is characterized by the presence of the 3,5-diprenyl-4-hydroxycinnamic acid artepillin C, together with other prenylated cinnamic acids and caffeic acid derivatives (Marcucci et al., 2001). Other propolis common constituents include organic acids, ketones, aldehydes, hydrocarbons, and minerals (Wagh, 2013).

### Antioxidant Activity

Propolis is the bee product containing the highest amount of phenolics and thus it has been deeply studied for antioxidant and radical scavenging activities (Viuda-Martos et al., 2008). Several of these compounds possess strong antioxidant and antiradicalic activities, including pinocembrin, chrysin, and pinobanksin (Sun et al., 2015). In DPPH and ORAC tests, pinobanksin-3-acetate has been indicated as the strongest antioxidant constituent (Boisard et al., 2014).

### Antimicrobial Activity

The antimicrobial activity of propolis has been demonstrated in clinical, *in vivo*, and *in vitro* studies. Propolis possesses antibacterial properties against Gram positive and negative strains, also validated in clinical trials (Noronha et al., 2014). Sensitive strains include MRSA, VRE, *Streptococcus* species, and *H. pylori* (Kosalec et al., 2005; Coelho et al., 2007).

Propolis antibacterial effects are possibly related to the presence of such flavonoids as galangin, pinocembrin, rutin, quercetin, and naringenin, as well as of CAPE, since these compounds are known to increase bacterial membrane permeability (Stepanovic et al., 2003). Inhibition of bacterial RNA polymerase has been also considered for galangin, pinocembrin, and CAPE (Speciale et al., 2006). The antimicrobial activity of Brazilian red propolis is thought to depend on its peculiar content in isoflavones (Freires et al., 2016).

Antibacterial effectiveness has been demonstrated for different propolis volatile fractions, including β-eudesmol and δ-cadinene in Bulgarian propolis, α-pinene and *trans*-β-terpineol in Greek propolis, β-eudesmol and benzyl benzoate in Hungarian propolis, nerolidol, spatulenol and ledol in Canary Island propolis, and farnesol, dihydroeudesmol and guaiol in Polish propolis (Bankova et al., 2014). The antibacterial activity of Brazilian propolis has been demonstrated for its volatile fractions containing nerolidol, spatulenol, p-cimen-8-ol, ethylphenol, β-caryophyllene, acetophenone, α-pinene, β-pinene and limonene (Bankova et al., 2014).

Propolis has been shown to act as an antifungal agent against pathogenic yeasts like *C. albicans*, *C. parapsilosis*, *C. tropicalis*, and *C. glabrata* (Al-Waili et al., 2012; Mutlu Sariguzel et al., 2016). Antifungal activity has been shown for volatile compounds from Brazilian propolis, viz. α-pinene, β-pinene and δ-cadinene, and from Turkish propolis, viz. phenyl-, ethyl-, and benzyl alcohol, and decanal (Ioshida et al., 2010). The ethanolic extracts of Iranian propolis have shown strong anti-*C. albicans* activity imputable to inhibition of germ tube development by phenolic, aromatic, and aliphatic acids (Haghdoost et al., 2016). Another ethanolic extract containing CAPE and other caffeic acid derivatives has been effective against *C. albicans, C. dubliniensis, C. glabrata, C. krusei, C. tropicalis*, and *C. parapsilosis*, with an MFC of 125-500 mg/L, while red Brazilian propolis rich in triterpenes and isoflavones, such as medicarpin, vestitol and formononetin, has shown the same MFC range (Freires et al., 2016).

### Antiviral Activity

Propolis is well known for its antiviral activity, which in some cases can exceed that of standard drugs. For instance, an ointment containing Canadian propolis has produced better results than acyclovir or placebo in the clinical treatment of genital herpes simplex (Vynograd et al., 2000). Antiviral properties seem to depend mainly on the presence of CAPE and related compounds. CAPE has been found to inhibit the activity of HIV-1 by acting on viral integrase (Costi et al., 2004), and to suppress hepatitis C virus replication *in vitro* (Zhang et al., 2003). Turkish Hatay propolis containing caffeic acid derivatives has been effective on herpes simplex virus 1 and 2 (Yildirim et al., 2016). 3,4-Dicaffeoylquinic acid, a major constituent of Brazilian green propolis, has repressed influenza A virus in mice by upregulating the TNF-related, apoptosis-inducing ligand (TRAIL) (Takemura et al., 2012).

### Immunomodulatory Activity

Propolis is generally known to modulate immune responses (Orsi et al., 2000), and this kind of effects could explain to some extent its antimicrobial and antiviral activities.

Brazilian green propolis standardized in 18.9% w/w polyphenols, 9.85% flavonoids and 2.3 artepillin C, administered to old mice, has enhanced phagocytosis, production of antibodies against sheep erythrocytes, and ear swelling (Gao et al., 2014). Brazilian green propolis and artepillin C have both inhibited *in vitro* alloreactive CD4^+^ T cell responses, together with the expression of IL-2, IL-17, and IFN-γ. In healthy subjects and asthmatic patients, CAPE has inhibited IL-5 and IFN-γ production, and the proliferation of CD4^+^ T cells stimulated by soluble anti-CD3 and anti-CD28 monoclonal antibodies, by targeting NF-κB and AKT/PKB pathways (Wang et al., 2010).

### Anti-inflammatory Activity

Many studies have reported anti-inflammatory properties of propolis, possibly linked to the presence of phenolic acids. CAPE is considered a particularly strong anti-inflammatory constituent, able to specifically target NF-κB signaling (Armutcu et al., 2015). This compound has been also found to modulate ERK MAPK signaling in T cells and mastocytes (Cho et al., 2014), and to regulate PI3K/Akt pathway in different human cell lines (Li et al., 2017). Possible downstream effects of these anti-inflammatory mechanisms may include the downregulation of key inflammatory enzymes, like xanthine oxidase, cyclooxygenase, matrix metalloproteinases, and inducible nitric oxide synthase (Armutcu et al., 2015; Li et al., 2017).

Anti-inflammatory virtues of propolis are popularly exploited in mouthwash products. Antigingivitis activity has been ascribed to phenolics, especially CAPE (Li et al., 2017). Moreover, in randomized, double-blind, placebo-controlled trials, rinse products containing Brazilian green propolis rich in artepillin C have alleviated gingivitis to the same extent of a NaF/cetylpyridinium chloride rinse or a chlorhexidine solution (Bretz et al., 2014).

Propolis is also lenitive to the skin by topical application. Australian and Romanian propolis have induced photo-protective effects on animal models, possibly due to the anti-UV properties of polyphenols (Cole et al., 2010; Bolfa et al., 2013).

### Wound Healing and Skin Protection

Animal models and clinical trials have shown the healing effect of propolis on diabetic foot ulcers and other problematic tissue injuries (Henshaw et al., 2014; Abu-Seida, 2015). Propolis wound healing activities are favored by the immunomodulatory, antioxidant and antiseptic effects of its rich phytocomplex (Martinotti and Ranzato, 2015). However, other mechanisms seem to play a role, since molecular studies have revealed that propolis modulates fibronectin expression and collagen I and III deposition in burns (Olczyk et al., 2013). An Indian propolis containing flavonoids, phenolic acids and terpenes, topically applied to rats with excision wounds, has upregulated hydroxyproline, hexosamine, uronic acid, nucleic acids and protein levels in wounded tissue, similar to the effect of nitrofurazone (Iyyam Pillai et al., 2010). In addition, in a study on Wistar rats, Brazilian green propolis rich in artepillin C has revealed superior wound healing activity with respect to Brazilian red propolis (Batista et al., 2012).

### Anticancer Activity

A very large number of *in vitro* and pre-clinical studies on propolis anticancer effects are available, while only few clinical studies have been conducted and their results are controversial. Propolis from Aydin, Turkey, rich in CAPE and flavonoids, has shown a concentration-dependent apoptotic effect on CCRF-SB lymphoblastic leukemic cells involving the modulation of different miRNA expressions (Yilmaz et al., 2016). New Zealand propolis and its constituents chrysin, galangin, CAPE, benzyl ferulate, benzyl isoferulate, pinostrobin, 5-phenylpenta-2,4-dienoic acid, and tectochrysin, have exhibited antiproliferative effects on DLD-1 colon cancer, HCT-116 colon carcinoma, KYSE-30 esophageal squamous cancer, and NCI-N87 gastric carcinoma cells (Catchpole et al., 2015). Polish propolis rich in phenolic acids and flavonoids has been shown to possess dose-dependent antiproliferative and proapoptotic activities on HCT 116 colon cancer and Me45 malignant melanoma cells (Kubina et al., 2015).

Various mechanisms of action have been disclosed for CAPE, including suppression of tyrosine kinase activity and induction of cell cycle arrest in G1 or G2/M phase (Patel, 2016), block of migration and invasiveness through Wnt inhibition and ROR2 upregulation (Tseng et al., 2016), voltage-gated sodium channel block leading to reduction of breast cancer cell motility and invasiveness (Fraser et al., 2016), and selective inhibition of cancerous cell viability (Kuo et al., 2015). Moreover, CAPE seems synergistic with tamoxifen on MCF-7 breast cancer cells (Motawi et al., 2016), and has induced radiosensitivity on MDA-MB-231 (estrogen receptor negative) and T47D (estrogen receptor positive) breast cancer cell lines (Khoram et al., 2016).

The flavone chrysin has exerted an antiproliferative effect on human Hep-3B, TCC, A549, HeLa, and colorectal cancer cells (Patel, 2016), while possible mechanisms of action include TRAIL-induced caspase activation and STAT3 inhibition (Lirdprapamongkol et al., 2013), as well as p38 and Bax activation (Pichichero et al., 2011). Artepillin C has induced an antiproliferative effect on prostate cancer cells by reducing TRAIL resistance and inhibiting NF-κB, while a proapoptotic effect of galangin has been related to the induction of MAPK phosphorylation (Zhang et al., 2013).

A study on M12.C3.F6 B-cell lymphoma cancer cell line has shown that pinobanksin, pinobanksin-3-O-propanoate, pinobanksin-3-O-butyrate and pinobanksin-3-O-pentanoate exert an antiproliferative effect by inducing loss of mitochondrial membrane potential and activating caspases 3, 8 and 9 (Alday et al., 2015). Similarly, the phenolic lipids cardanol and cardol from Thai propolis have shown antiproliferative effects on several human cancer cells (Teerasripreecha et al., 2012), while cardanol has also induced apoptosis in BT-474 breast cancer cells, upregulated p21, stimulated ERK, p38 and JNK phosphorylation, and downregulated cyclin D (Buahorm et al., 2015). Finally, the polycyclic, polyisoprenylated benzophenone nemorosone from Cuban propolis has shown anticancer effects on estrogen receptor-positive MCF-7 cells by blocking cell cycle in G0/G1, and reducing MAPK and Akt phosphorylation (Popolo et al., 2011).

### Adverse Effects

Clinical and *in vivo* studies on animal models have reported that propolis is well tolerated and non-toxic. The No Observed Adverse Effect Level (NOAEL) on mice and rats is over 1,470 mg/Kg/day at 60 days, and over 2,470 mg/Kg/day at 90 days (Burdock, 1998). In humans, toxic effects occur at dosages as high as 15 g/die (Castaldo and Capasso, 2002). However, despite its favorable safety profile, propolis is a common cause of allergic reactions. It has been reported that 1.2–6.6% of patients with dermatitis are sensitive to propolis (Walgrave et al., 2005), while major sensitizers are 3-methyl-2-butenyl caffeate, phenylethyl caffeate, benzyl salicylate, benzyl cinnamate, and 1,1-dimethylallylcaffeic acid (Burdock, 1998; Walgrave et al., 2005).

## Bee Venom

Bee venom, also known as apitoxin, is a complex fluid secreted by the bee venom gland located in the abdominal cavity and injected into victims by a stinger, causing local inflammation, anticoagulant effect, and immune response. Bee venom constituents include amphipathic polycationic peptides, of which major ones are melittin and apamin, enzymes such as phospholipase A2, and low-molecular weight compounds including active bioamines such as histamine and catecholamines (Lee et al., 2016).

The venom has been traditionally, used in acupuncture and apitherapy, consisting in its injection to the patient as analgesic, against chronic pain and inflammation, and for other purposes such as immunotherapy and Parkinson’s treatment. A number of anticancer effects have been reported, together with antimutagenic, antinociceptive, and radioprotective properties. Approved pharmaceutical use has been introduced, while attempts at validating clinical treatment for chronic pain have been made (Moreno and Giralt, 2015; Sobral et al., 2016). However, different bee venom constituents are allergenic and in hypersensitive people bee sting can arrive to produce fatal outcome (Gelder et al., 1996).

### Melittin

Melittin is a peptide of 26 amino acid residues, with a prevalently hydrophobic N-terminus and a hydrophilic C-terminus. It has distinct biological activities and has attracted much interest from pharmacological and biotechnological points of view.

The toxicity mechanism consists in the disruption of phospholipid bilayers, leading to cell lysis and the release of tissue injurious compounds such as lysosomal enzymes, serotonin, and histamine, triggering inflammation and pain (Raghuraman and Chattopadhyay, 2007). Together with hyaluronidase and phospholipase A2, melittin is responsible for venom allergenic properties. It seems also a major cause of pain induction by the bee venom, through activation of TRPV receptors and release of algogens from injured cells (Chen et al., 2016). In contrast to its toxicity, melittin is known as a traditional anti-inflammatory remedy for various diseases, such as dermatitis, neuritis, liver inflammation, atherosclerosis, and arthritis, but the mechanism of action at the cellular level has not been clarified (Lee and Bae, 2016). A possible coherent mechanism of antiatherosclerotic effects of melittin consists in the inhibition of vascular smooth muscle proliferation through the hindrance of platelet-derived growth factor beta-receptor signaling (Son et al., 2007).

The ability of interacting with biological membranes confers strong antimicrobial properties to melittin, which have attracted interest for fighting human pathogens, such as methicillin-resistant *S. aureus* (Choi et al., 2015), as well as plant pathogens (Stockwell and Duffy, 2012). Anticancer activities of melittin have been reported by different sources, while attempts at clarifying molecular mechanisms have been made by *in vitro* studies (Gajski and Garaj-Vrhovac, 2013). It has been shown for instance that melittin induces apoptosis in human ovarian cancer cells, SKOV3 and PA-1, by increasing the expression levels of the DR3, DR4, and DR6 death receptors (Jo et al., 2012).

Despite many indications of possible therapeutic applications of melittin, *in vivo* injection is known to entails side effects like hemolysis and liver injury, which have stimulated studies for the development of non-toxic hybrid derivatives. Engineered melittin peptides have also been developed for different biotechnological applications, e.g., to enhance antimicrobial properties or promote siRNA release from endosomes into target cells. (Moreno and Giralt, 2015).

### Apamin

Apamin is a peptide of 18 amino acids, tightly cross-linked by the presence of two disulphide bonds (Habermann, 1984). It exerts a highly specific toxicity mechanism, consisting in a block of small conductance Ca^2+^-dependent K^+^ channels (SK channels) expressed in the central nervous system and in other districts, like the cardiovascular system and smooth muscle (Adelman et al., 2012).

Due to its ability of selectively targeting SK channels, apamin has been used as a tool for the physiological characterizations of this kind of K^+^ conductance (Castle et al., 1989). On a pharmacological ground, such a property has been adopted as an explanatory paradigm for accumulating evidence that apamin facilitates learning and memory. Apamin can cross the blood-brain barrier, and its administration to animals improves cognitive deficits, suggesting that SK channels would be appropriate apamin targets in the treatment of these neural disorders (Deschaux and Bizot, 2005; Brennan et al., 2008). In addition, the possibility of using apamin or less toxic analogs as blood-brain barrier, drug-delivery shuttles has been explored (Oller-Salvia et al., 2013).

SK channels are known to be involved in the pathogenesis of Parkinson’s disease. Consistent with this premise, another important perspective for neuro-therapeutic uses of apamin derives from its ability of protecting dopaminergic neurons from degeneration in experimental models of Parkinson’s (Alvarez-Fischer et al., 2013; Thomas and Justin, 2013). Among other possible uses, experimental work has shown antiatherosclerotic effects of apamin administered to mice (Kim et al., 2012), while as a K^+^ channel blocker, apamin can be useful for long-term whole blood storage (Delgado and Pitt, 2008).

### Phospholipase A2

Phospholipase A2 (PLA2) hydrolyzes complex lipids to produce a fatty acid and various reaction products, including lysophosphatidic acid, lysophosphatidylcholine, and sphingosine phosphate. These latters exert cytotoxic and immunostimulatory effects on various cell types, eventually triggering immune responses and inflammation.

Phospholipase A2 is the major allergen of bee venom, containing three peptide and one glycopeptide T cell epitopes recognized by allergic and non-allergic subjects (Dhillon et al., 1992; Okano et al., 1999). However, PLA2 has also properties translatable into therapeutic treatments. It has exerted neuroprotective effects in a mouse model of Parkinson’s disease by activating regulatory T lymphocytes (Treg) that are known to mediate peripheral immune tolerance (Chung et al., 2015). Systemic PLA2 administration to a mouse model of neuropathic pain has alleviated cold and mechanical allodynia through the activation of α2-adrenegic receptors (Li et al., 2015). It has also been shown that PLA2 acts cooperatively with phosphatidylinositol-(3,4)-bisphosphate in inducing *in vitro* lysis of different tumor cell lines (Putz et al., 2006).

### Minor Peptides and Enzymes

The second major allergen of honeybee venom is hyaluronidase (Padavattan et al., 2007), while other allergenic peptides include icarapin isolated from *A. cerana* (Wong et al., 2012), and two serine proteases named Api SI and Api SII belonging to the prophenoloxidase activating factor II family (Georgieva et al., 2011). Another serine protease of this family, named Bi-VSP, has a dual behavior, since in arthropods it triggers the phenoloxidase cascade inducing a lethal immune response, while in mammals it acts as a toxic thrombin-like and plasmin-like fibrinolytic protease (Choo et al., 2010).

Secapin is a serine protease inhibitor-like peptide exerting anti-fibrinolytic and anti-elastolytic activities, and also displaying antimicrobial properties by binding to the surfaces of fungi and bacteria (Lee et al., 2016). Two peptides isolated from *A. cerana* venom, viz. an inhibitor cysteine knot (ICK) peptide and a Kazal-type serine protease inhibitor, have been shown to act as antibacterial, antifungal and insecticidal venom toxins (Kim et al., 2013; Park et al., 2014).

Tertiapin is a 21 amino acid neurotoxin blocking inward-rectifier K^+^ channels expressed in epithelial cells, heart, and central nervous system. In the heart, tertiapin contrasts G-protein-gated, acetylcholine-activated K^+^ current that mediate parasympathetic heart rate decrease. This toxin could be allegedly useful as a drug for treating disorders in atrio-ventricular transmission, but at present it is used solely as a tool for K^+^ channel modulation (Drici et al., 2000).

Mast cell degranulating (MCD) peptide is a 22-amino acid peptide with two disulfide bridges, structurally similar to apamin but with different mechanisms of action. At low concentrations, MCD induces mast cell degranulation through histamine release, while at higher concentrations it can produce anti-inflammatory effects (Buku, 1999). Moreover, MCD also acts as a neurotoxin by blocking fast-inactivating (A-type) and slow-inactivating (delayed rectifier) K^+^ channels, thereby increasing neuronal excitability. Long term potentiation in the hippocampus CA1 region has been experimentally observed, while direct brain injection leads to convulsions and neurodegeneration (Mourre et al., 1997).

## Bee Pollen

Foraging bees bring pollen back to the hive where it is packed into pellets and stored. During this process, the pollen mixed with nectar and bee salivary secretions becomes the “bee bread,” representing a main food reserve for the hive colony (Almeida-Muradian et al., 2005).

Main chemical compounds of bee pollen include carbohydrates, proteins and amino acids, lipids and fatty acids, phenolics, enzymes and coenzymes, vitamins and minerals (Komosinska-Vassev et al., 2015). However, the chemical composition of bee pollen is highly variable, depending on plant source, geographical region, and climatic conditions, thus deeply affecting biological properties and therapeutic virtues (Denisow and Denisow-Pietrzyk, 2016).

Bee pollen is an energy food used by humans as a diet supplement and for the conditioning of athletes. The high content of protein, fat, and minerals (particularly Ca, Mg, Fe, and P) gives bee pollen a nutritional value similar to, or higher than, that of dried legumes. Among vitamins, the levels of pantothenic and nicotinic acids are close to those of beef, ascorbic acid is similar to that of vegetables, such as lettuce and tomatoes, and riboflavin is comparable to that of skimmed milk power (Linskens and Jorde, 1997).

Bee pollen is used in complementary and alternative medicine to cure prostatitis, stomach ulcers, infectious diseases, and for the prevention and treatment of high-altitude-sickness syndrome (Linskens and Jorde, 1997). A wide range of therapeutic properties have been suggested, including antimicrobic, antioxidant, hepatoprotective, chemopreventive and anticarcinogenic, antiatherosclerotic, anti-inflammatory, antiallergenic, and immunomodulatory activities (Komosinska-Vassev et al., 2015; Denisow and Denisow-Pietrzyk, 2016).

### Antioxidant Activity

The antioxidant activity of bee pollen seems to be mainly due to phenolic acids, like vanillic, protocatechuic, gallic, and *p*-coumaric acids, and to flavonoids like hesperidin, rutin, kaempferol, apigenin, luteolin, quercetin, and isorhamnetin. These compounds are thought to inactivate electrophiles and scavenge free radicals and reactive oxygen species (Bonvehí et al., 2001; Pascoal et al., 2014).

### Antimicrobial Activity

Antimicrobial effects of bee pollen are well known, possibly mediated by glucose oxidase activity, deriving from honeybee secretion, while plant phenolics and flavonoids could also be involved (Denisow and Denisow-Pietrzyk, 2016; Fatrcova-Sramkova et al., 2016). Evidence about the activity of phenolic compounds from bee pollen extracts against Gram-positive and Gram-negative pathogenic bacteria, microscopic fungi and yeasts, has been reported (Baltrušaitytė et al., 2007; Kacániová et al., 2012).

### Anti-inflammatory Activity

Bee pollen exerts anti-inflammatory effects that have been compared to those of common non-steroidal anti-inflammatory drugs, possibly depending on the activity of flavonoids, phenolic acids, phytosterols, and flavoring substances like anethole, an inhibitor of the NF-*K*B pathway (Middleton, 1998; Choi, 2007). Specific effects include the capability of removing swellings caused by cardiovascular and renal pathologies (Yakusheva, 2010), of protecting the liver from carbon tetrachloride-induced damages (Yildiz et al., 2013), and of alleviating prostate inflammation and hyperplasia (Yakusheva, 2010). Positive effects on prostatic conditions have been also ascribed to antiandrogen actions (Rzepecka-Stojko et al., 2012).

### Anticancer Activity

Different studies have shown potential anticancer activity of bee pollen, probably associated with antioxidant and antimutagenic potentials (Denisow and Denisow-Pietrzyk, 2016). The steroid fraction of a chloroform extract from *Brassica campestris* bee pollen has shown strong cytotoxicity on human prostate cancer PC-3 cells, associated to stimulation of TNF-α secretion and apoptosis induction (Wu and Lou, 2007).

### Antiatherosclerotic and Antidiabetic Activities

Bee pollen has shown antiatherosclerotic and cardioprotective effects and has been successfully applied to patients who did not respond to classical drugs (Polanski et al., 1998). Hypolipidemic activity, confirmed by pharmacological studies conducted on rats and rabbits, has been ascribed to the presence of unsaturated fatty acids, especially the ω-3, α-linolenic acid, and to phospholipids and phytosterols (Komosinska-Vassev et al., 2015). α-Linolenic acid is a precursor of prostaglandin-3a that is considered a major inhibitor of platelet aggregation (Denisow and Denisow-Pietrzyk, 2016).

Ghoshal and Saoji () have found the presence of antidiabetic compounds in pollen grains, such as steroids and alkaloids in the pollen of *C. roseus*, saponins, flavonoids, sugars, and tannins in *M. charantia*, sugars, flavonoids and sterols in *B. monosperma*, and alkaloids and tannins in *S. cuminii*, suggesting therapeutic possibilities for bee pollen as a hypoglycemic agent.

### Immunomodulatory Activity

Evidence of antiallergic activity of bee pollen has been reported, including prevention of IgE binding to their high-affinity receptor Fc*ε*RI, inhibition of histamine release from mast cells, and basophil degranulation (Ishikawa et al., 2008; Moita et al., 2014). Flavonoids, steroids, and volatile oil compounds seem to be involved in immunosuppressive activities.

### Nutritional Properties

Bee pollen has been used as a diet supplement in recovery periods, in cases of malnutrition, asthenia and apathy, and to increase physical and mental ability or strengthen the immune system. Experiments on animals have shown that the administration of bee pollen prolongs life span, promotes weight gain, increases plasma hemoglobin levels, and provides tissues with vitamin C and Mg (Khalil and El-Sheikh, 2010; Attia et al., 2011). These virtues may be related to a complex of active substances, including amino acids, vitamins like tocopherol, niacin, thiamine, biotin, and folic acid, polyphenols, carotenoids, phytosterols, and minerals (Denisow and Denisow-Pietrzyk, 2016).

### Adverse Effects

Health risks linked to the use of bee pollen may derive from the occasional presence of contaminants such as heavy metals, pesticides, mycotoxins (e.g., ochratoxin A), and bacteria (Denisow and Denisow-Pietrzyk, 2016). Moreover, bee pollen derived from *Echium vulgare, Symphytum officinale*, and *Senecio jacobaea* may contain dangerous levels of pyrrolizidine alkaloids with hepatotoxic properties (Kempf et al., 2010).

As known, pollen is highly allergenic, and consequently, complications or anaphylaxis due to bee pollen use have been reported, making tests for individual sensitivity highly recommendable before use (Jagdis and Sussman, 2012).

## Beeswax

Worker bees secrete beeswax by wax glands located in abdominal segments. This substance is generally produced in greatest amount during colony growth phase in late spring, and is used for making combs. Beeswax is synthesized starting from honey sugars, and has a crystalline structure suitable for hive construction.

Chemical composition varies among bee species and geographical zones, and includes hydrocarbons, of which major ones are heptacosane, nonacosane, hentriacontane, pentacosane and tricosane, free fatty acids and free fatty alcohols, linear wax monoesters, hydroxymonoesters deriving from palmitic, 15-hydroxypalmitic, and oleic acids, and complex wax esters containing 15-hydroxypalmitic acid and diols (Münstedt and Bogdanov, 2009). A total of about 50 aroma components has also been reported (Ferber and Nursten, 1977). The ester/acid ratio is important for beeswax characterization by different Pharmacopeias, being generally lower (3–4) in European, and higher (8–9) in Asian beeswax (Münstedt and Bogdanov, 2009).

Beeswax is used as an additive in a variety of industrial products and processes, such as food industry, candles, and cosmetics. In pharmaceutical preparations it plays a role as a thickener, binder, drug carrier and release retardant.

### Antimicrobial Activity

Beeswax has been used since ancient times for its antimicrobial properties in European and Asian traditional medicines. Preservative effects are possibly at the basis of its use in embalming and mummification practices by old Egyptians and Persian, or to model death masks by ancient Romans.

A beeswax crude extract has shown inhibitory effects against *S. aureus, Salmonella enterica, C. albicans* and *Aspergillus niger* (Ghanem, 2011), while effects against pathogenic bacteria and microscopic fungi have been reported for methanol and ethanol extracts (Kacániová et al., 2012). This kind of effects could depend at least in part on beeswax compounds of plant origin (Puleo and Keunen, 1991).

### Dermatological and Cosmetic Properties

Beeswax has been known as a major Ayurvedic remedy for inflammation, bruises, burns, and cracked heels (Gokani, 2014). Ointments based on beeswax useful for joint pain, wounds and burns, are reported in the Ebers Papyrus (about 3500 B.P.), by the Greek-Roman physician Galen (about 2150 B.P.), and in old texts of traditional Chinese medicine, such as the “Shen Nong Book of Herbs” (about 2100–2200 B.P.) (Rit and Behrer, 1999).

Thanks to its very low irritant and comedogenic effects, beeswax is widely used in modern cosmetics and makeup as a thickener, emollient and emulsifier (Münstedt and Bogdanov, 2009).

## Future Perspectives

Pharmaceutical and clinical uses of honeybee products are attracting increasing interest. Research and development in this field generally concern whole materials or subfractions rather than single compounds. Clinical trials have been conducted, among others, with honey for cicatrization problems and diabetes, with royal jelly for diabetes and rheumatoid arthritis, with propolis for disinfection and gingivitis, and with bee venom for Parkinsons’ and rheumatoid arthritis^1^. However, the complexity and variability in composition of these products raise the need of their standardization before safe and predictable clinical uses can be achieved.

Conversely, the use of specific compounds from honeybee products as curative drugs has not been implemented yet. As shown in the present review, various of these compounds have been in-depth characterized for their effects on biochemical pathways, cells, and organs, suggesting a series of possible uses as therapeutic drugs (**Table 1**). Moreover, preclinical studies indicate that some of these agents may be competitive with standard drugs, possibly including MGO, MRJPs, jelleins, royalisin, 10-HDA, CAPE, artepillin C, melittin, and apamin. However, none of them is reported at the online clinical trial database https://clinicaltrials.gov/, with the exception of CAPE^2^, suggesting that their medicinal use as specific drugs is not forthcoming.

**Table 1:** Reported effects for major compounds from different honeybee products.

Product	Compound	Effect	Reference
Honey	H_2_O_2_	Antimicrobial activity	Brudzynski et al., 2011
	MGO	Antimicrobial activity	Mavric et al., 2008
	Defensin-1	Antimicrobial activity	Kwakman and Zaat, 2012
	Trihydroxyketone	Apoptosis induction and p65, NF-KB and IL-6 repression in PC-3 cancer cells	Kassi et al., 2014
Royal Jelly	MRJP-1	Block of mannose receptors in human phagocytic cells	Molan and Rhodes, 2015
	MRJP-1	Induction of TNF-α and MMP-9 in keratinocytes	Majtan et al., 2010
	MRJP-1	Increase of fecal bile acids and cholesterol excretion	Kashima et al., 2014
	MRJP-1 oligomer	Promotion of Jurkat lymphoid cell proliferation	Moriyama et al., 2015
	MRJP-1 and MRJP-2	Stimulation of TNF-α release from macrophages	Majtan et al., 2006
	MRJP-3	Suppression of IL-2, IL-4, and IFN-γ production by antigen-stimulated T cells	Okamoto et al., 2003
	Apalbumin 2a (MRJP-2 variant)	Antimicrobial activity	Bilikova et al., 2009
	Jelleins	Antimicrobial activity	Fontana et al., 2004
	Royalisin	Antimicrobial activity	Bachanova et al., 2002
	Royalactin	Increase of lifespan in honeybees and other invertebrates	Detienne et al., 2014
	10-HDA	Antimicrobial activity	Alreshoodi and Sultanbawa, 2015
	10-HDA	Inhibition of LPS-induced NF-κB activation in murine macrophages	Sugiyama et al., 2012
	10-HDA	Inhibition of histone deacetylase	Makino et al., 2016
	10-HDA	Inhibition of synoviocytes from rheumatoid arthritis	Wang et al., 2015
	10-HDA	Inhibition of IL-12 production by dendritic cells and of NO production by macrophages	Sugiyama et al., 2013
	10-HDA	Modulation of dendritic cell, Th1 and Th2 activity	Mihajlovic et al., 2013
	10-HDA	Increase of insulin-independent muscle glucose uptake	Takikawa et al., 2013
	10-HDA	Improvement of hyperlipidemic condition in rats	Xu et al., 2002
	10-HDA	Increase of longevity and oxidative stress tolerance in *Caenorhabditis elegans*	Honda et al., 2015
	10-HDA	Increase of TGF-β1production and collagen synthesis in fibroblasts	Park et al., 2011
	10-HDA	Inhibition of MMP-1 and MMP-3 release from rheumatoid arthritis fibroblasts	Yang et al., 2010
	10-HDA	Prevention of UVA-induced JNK/p38 activation and MMP-1 and MMP-3 upregulation in fibroblasts	Zheng et al., 2013
	10-HDA and 10-hydroxydecanoic	Inhibition of TRPA1 and TRPV1 receptors	Terada et al., 2011
	10-HDA	Stimulation of neuron differentiation from rat embryo stem cells	Hattori et al., 2007
	3,10-dihydroxydecanoic acid	Stimulation of dendritic cell differentiation	Dzopalic et al., 2011
	Sebacic acid	Antifungal activity	Melliou and Chinou, 2005
Propolis	CAPE	Inhibition of HIV-1 integrase	Costi et al., 2004
	CAPE	Suppression of hepatitis C virus replication *in vitro*	Zhang et al., 2003
	CAPE	Antigingivitis activity	Li et al., 2017
	CAPE	Inhibition of IL-5 and IFN-γ production	Wang et al., 2010
	CAPE	Inhibition of CD4^+^ T cell proliferation by targeting NF-κB and AKT/PKB	Wang et al., 2010
	CAPE	Anti-inflammatory effect by targeting NF-κB	Armutcu et al., 2015
	CAPE	Modulation of ERK in T cells and mastocytes	Cho et al., 2014
	CAPE	Regulation of PI3K/AKT pathway in human cells	Li et al., 2017
	CAPE	Suppression of tyr kinase activity and induction of cell cycle arrest	Patel, 2016
	CAPE	Block of cancer cell migration and invasiveness	Tseng et al., 2016
	CAPE	Voltage-gated sodium channel block in breast cancer cells	Fraser et al., 2016
	CAPE	Selective inhibition of cancerous cell viability	Kuo et al., 2015
	Artepillin C	Inhibition of CD4^+^ T cells, and of IL-2, IL-17, and IFN-γ release	Wang et al., 2010
	Artepillin C	Antiproliferative effect on prostate cancer cells by targeting NF-κB	Zhang et al., 2013
	Chrysin, galangin, CAPE, benzyl ferulate, benzyl isoferulate, pinostrobin, 5-phenylpenta-2,4-dienoic acid, and tectochrysin	Antiproliferative activity on different cancer cell lines	Catchpole et al., 2015
	Chrysin	Antiproliferative effect on different cancer cells	Patel, 2016
	Chrysin	TRAIL-induced caspase activation and STAT3 inhibition in cancer cells	Lirdprapamongkol et al., 2013
	Chrysin	p38 and Bax activation in cancer cells	Pichichero et al., 2011
	Galangin, pinocembrin, and CAPE	Inhibition of bacterial RNA polymerase	Speciale et al., 2006
	Galangin	Proapoptotic effect on cancer cells	Zhang et al., 2013
	Pinobanksin	Loss of mitochondrial membrane potential and caspase activation in cancer cells	Alday et al., 2015
	Pinobanksin-3-acetate	Strong antioxidant effect	Boisard et al., 2014
	3,4-dicaffeoylquinic acid	TRAIL upregulation and repression of influenza A virus in mice	Takemura et al., 2012
	Cardanol and cardol	Antiproliferative effect on different cancer cells	Teerasripreecha et al., 2012
	Cardanol	Induction of apoptosis in breast cancer cells with cyclin D downregulation	Buahorm et al., 2015
	Nemorosone	Inhibition of MCF-7 cells by cell cycle block and of MAPK and AKT	Popolo et al., 2011
Bee venom	Melittin	Cytotoxic effect by disruption of phospholipid bilayers	Raghuraman and Chattopadhyay, 2007
	Melittin	Activation of TRPV receptors and release of algogens	Chen et al., 2016
	Melittin	Anti-inflammatory effect	Lee and Bae, 2016
	Melittin	Inhibition of PDGFR-β signaling	Son et al., 2007
	Melittin	Antimicrobial effect	Choi et al., 2015
	Melittin	Apoptosis induction in ovarian cancer cells by DR3, DR4, DR6 upregulation	Jo et al., 2012
	Apamin	Block of SK channels	Castle et al., 1989
	Apamin	Antiatherosclerotic effect in mice	Kim et al., 2012
	PLA2	Allergenic effect	Okano et al., 1999
	PLA2	Treg activation in Parkinson’s mouse model	Chung et al., 2015
	PLA2	Activation of α2-adrenegic receptors in allodynia mouse model	Li et al., 2015
	Hyaluronidase	Allergenic effect	Padavattan et al., 2007
	Bi-VSP serine protease	Thrombin-like and plasmin-like fibrinolysis	Choo et al., 2010
	Secapin	Serine protease inhibition and antimicrobial effect	Lee et al., 2016
	Inhibitor cysteine knot peptide and Kazal-type serine protease inhibitor	Antimicrobial and insecticidal effects	Park et al., 2014
	Tertiapin	Inward-rectifier K^+^ channel block	Drici et al., 2000
	MCD	Mast cell degranulation and histamine release	Buku, 1999
	MCD	Fast- and slow-inactivating K^+^ channel block	Mourre et al., 1997
Bee Pollen	Anethole	NF-*K*B pathway inhibition	Choi, 2007

*MGO, methylglyoxal; MRJP, major royal jelly protein; 10-HDA, 10-hydroxy-2-decenoic acid; CAPE, caffeic acid phenethyl ester; PLA2, phospholipase A2; MCD, mast cell degranulating peptide*.

Different reasons may explain the gap between the clinical exploitation of whole honeybee products and their single constituents. A role may be played in some cases by the toxicity of the bioactive agent, or alternatively, by an economically inefficient scale-up of pharmaceutical manufacturing, even though some compounds can be obtained by synthetic way too. Procedures for the chemical synthesis of MGO and CAPE have been patented, while synthetic MGO has been added in some cases to medicinal honey for strengthening wound healing properties (Wardell and Sabacinski, 2016). Methods for 10-HDA purification from royal jelly, artepillin C from propolis, melittin and apamin from bee venom, and their uses in pharmaceutical preparations have been also patented (Iinuma et al., 2014). Regardless whether the pharmaceutical exploitation of these bioactives is going to take off or not, studies aimed at refining the knowledge of their mechanisms of action remain of pivotal importance for developing applications of honeybee products to medicinal uses.

## Author Contributions

LC contributed with ethnobotanical and pharmacognosy aspects; MB contributed with pharmaceutical aspects; JX contributed with chemical aspects; BB contributed with aspects on mechanisms of actions.

## Conflict of Interest Statement

The authors declare that the research was conducted in the absence of any commercial or financial relationships that could be construed as a potential conflict of interest.

## Footnotes

1 https://clinicaltrials.gov

2 https://clinicaltrials.gov/ct2/show/NCT02744703?term=caffeic+acid\&rank=3
